# Arp2/3 Complex Regulates Asymmetric Division and Cytokinesis in Mouse Oocytes

**DOI:** 10.1371/journal.pone.0018392

**Published:** 2011-04-08

**Authors:** Shao-Chen Sun, Zhen-Bo Wang, Yong-Nan Xu, Seung-Eun Lee, Xiang-Shun Cui, Nam-Hyung Kim

**Affiliations:** 1 Department of Animal Sciences, Chungbuk National University, Cheongju, Republic of Korea; 2 State Key Laboratory of Reproductive Biology, Institute of Zoology, Chinese Academy of Sciences, Beijing, China; Baylor College of Medicine, United States of America

## Abstract

Mammalian oocyte meiotic maturation involves oocyte polarization and a unique asymmetric division, but until now, the underlying mechanisms have been poorly understood. Arp2/3 complex has been shown to regulate actin nucleation and is widely involved in a diverse range of processes such as cell locomotion, phagocytosis and the establishment of cell polarity. Whether Arp2/3 complex participates in oocyte polarization and asymmetric division is unknown. The present study investigated the expression and functions of Arp2/3 complex during mouse oocyte meiotic maturation. Immunofluorescent staining showed that the Arp2/3 complex was restricted to the cortex, with a thickened cap above the meiotic apparatus, and that this localization pattern was depended on actin. Disruption of Arp2/3 complex by a newly-found specific inhibitor CK666, as well as by Arpc2 and Arpc3 RNAi, resulted in a range of effects. These included the failure of asymmetric division, spindle migration, and the formation and completion of oocyte cytokinesis. The formation of the actin cap and cortical granule-free domain (CGFD) was also disrupted, which further confirmed the disruption of spindle migration. Our data suggest that the Arp2/3 complex probably regulates oocyte polarization through its effect on spindle migration, asymmetric division and cytokinesis during mouse oocyte meiotic maturation.

## Introduction

Oocyte polarization results in a unique asymmetric division. The oocyte is transformed into a highly polarized MII-arrested egg during mammalian meiotic maturation, which is essential to allow asymmetric division and the retention of the maternal components for early development [1]. Disruption of this asymmetry usually occurs in oocytes that are of low quality or that have experienced post-ovulatory ageing. Oocyte polarization, which includes spindle migration, spindle anchoring and cortical reorganization, as well as asymmetric division, is controlled by microtubule and microfilament cytoskeletons [2], [3]. After GVBD (GV breakdown), the centrally positioned spindle translocates to the cortex of the oocyte in an actin-dependent way. Furthermore, cortical granules (CGs) are redistributed to form a CG-free domain (CGFD), microvilli are lost in the region overlaying the spindle, and microfilaments are enriched to form an actin cap [4], [5], [6]. Together, these changes are referred to as cortical reorganization and polarization. When cortical polarity becomes intense, the oocyte extrudes the polar body, leaving a highly polarized egg.

Unlike common ligand-mediated cell polarity, the development of oocyte polarity and cortical reorganization is independent of the involvement of any external ligand as the signal is intrinsic to the oocyte [7]. Meanwhile, meiotic spindles in oocytes lack true centrosomes, indicating that specialized mechanisms may be responsible for the off-centre positioning of the spindles. Until now, the molecular details of oocyte polarization have been poorly understood.

Arp2/3 complex (actin-related protein 2/3 complex) consists of Arp2, Arp3 and five other subunits; Arpc1 to Arpc5 [8], [9]. The complex binds to the side of an existing actin filament and initiates the new filament assembly [8]. ARP2 and ARP3 are actin-related proteins that nucleate the growth of the new filament, and the other five proteins link the two actin-related proteins to the mother filament [10]. Arp2/3 complex is involved in a range of cellular processes. In many species, inhibition of the activity of the complex by RNAi or inhibitory antibodies results in the disruption of cell migration and adhesion [11], [12], [13], endocytosis [14], [15], and the establishment of cell polarity during mitosis (see reviews [8], [10]). The involvement of Arp2/3 complex in the formation of new branched actin filaments is dependent upon interactions with nucleation-promoting factors (NPFs). The NPFs consist of WASP [16], N-WASP [17], [18], WAVE1 [19], [20], WAVE2 [21], [22], WAVE3, and the newly identified compounds, WASH [23], WHAMM [24] and JMY [25]. Recent work has demonstrated that Abp1 [26], Pan1 and cortactin [27], [28], [29] also activate the Arp2/3 complex, whilst the NPFs are activated by Cdc42 and Rac [30], [31], [32].

Recent studies using mouse oocytes have shown that the activators of Arp2/3, Cdc42 and Rac are necessary for oocyte polarization, spindle formation and migration during meiosis [33], [34], [35]. The current study investigated whether the Arp2/3 complex is involved in oocyte polarization during oocyte meiotic maturation. The complex was found to show a unique expression pattern and its inhibition by a specific inhibitor and RNAi demonstrated that it is indeed involved in this process and the resulting asymmetric division.

## Results

### Localization of the Arp2/3 complex during mouse oocyte meiotic maturation

**Figure 1:**
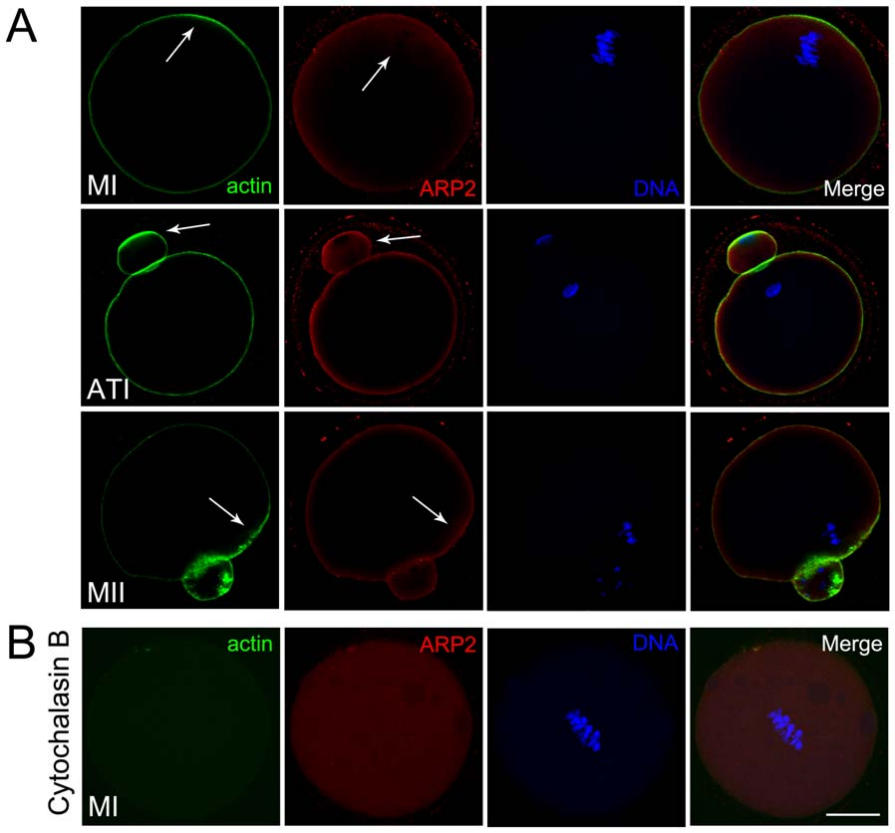
Localization of Arp2/3 complex in mouse oocytes. (**A**) Subcellular localization of the Arp2/3 complex during mouse oocyte meiotic maturation. ARP2 antibody staining was employed to show the subcellular localization of the Arp2/3 complex in mouse oocytes as revealed by immunofluorescence staining. From the GV to the MII stage, all ARP2 accumulated at the cortex of the oocytes and the region near the cortex. Green, actin; red, ARP2; blue, chromatin. Bar = 20 µm. (**B**) Subcellular localization of the Arp2/3 complex after CB treatment during mouse oocyte meiotic maturation. The subcellular localization of ARP2 in mouse oocytes was revealed by immunofluorescence staining. Actin was disrupted during the MI stage and ARP2 dispersed into the cytoplasm. Green, actin; red, ARP2; blue, chromatin. Bar = 20 µm.

The subcellular localization of the Arp2/3 complex at different stages of meiotic maturation was examined by ARP2 antibody immunofluorescent staining. As shown in Fig. 1A, actin localized at the cortex of the oocytes, and an actin cap formed in the area overlying the chromosomes during the late MI stage and in the ATI and MII stages. During these stages, ARP2 was concentrated primarily in the cortex of the oocyte where it co-localized with actin. We also found ARP2 to be enriched in the actin cap and to have a greater distribution around the chromosomes.

### Localization of ARP2 and actin after cytochalasin B treatment

The relationship between the Arp2/3 complex and actin dynamics was investigated through the successful disruption of F-actin by cytochalasin B treatment. ARP2 was found to disperse to the cytoplasm and no specific localization pattern was observed in MI stage oocytes (Fig. 1B).

### CK666 treatment and RNAi cause disruption of asymmetric division

**Figure 2:**
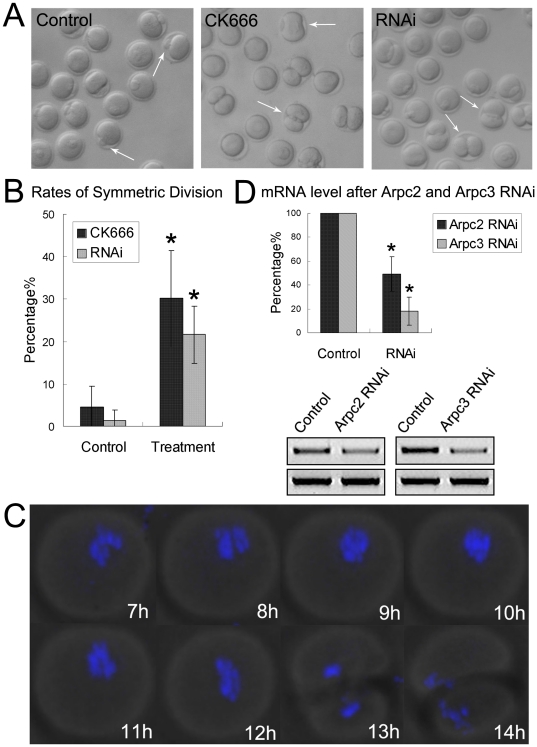
Effects of CK666 and RNAi treatment on asymmetric division in mouse oocytes. (**A**) Oocytes underwent symmetric division after treatment with CK666 and RNAi. (**B**) Rates of symmetric division when the oocytes were cultured with CK666 in M16 medium, and after RNAi. (**C**) Time lapse microscopy of maturing oocytes treated with CK666 after GVBD. These oocytes divided symmetrically. (**D**) Levels of Arpc2 and Arpc3 mRNA after Arpc2 and Arpc3 siRNA injection. *, significantly different (p<0.05).

To further investigate the roles of the Arp2/3 complex during mouse oocyte meiotic maturation, we employed the newly-found Arp2/3 specific inhibitor, CK666 [36]. CK-666 targets a pocket formed between Arp2 and Arp3, preventing the complex from shifting into an active conformation. As shown in Fig. 2A, after 12 h in culture, most oocytes (78.7±4.8%, n = 219) extruded the first polar body normally in the control group, but a large proportion of those in the CK666 treatment group underwent symmetric division, producing two cells of a similar size (30.2±11.3%, n = 123 vs 4.5±5%, n = 120) (p<0.05) (Fig. 2B). We next employed time-lapse microscopy to examine the dynamic changes occurring in maturing oocytes and further confirmed that they tended to divide equally after 12 h in culture (Fig. 2C).

Arpc2 and Arpc3 siRNA injection was used to down-regulate the expression of the Arp2/3 complex, which successfully depressed the mRNA level of both Arpc2 and Arpc3 (49.2±14.4% vs 100%; 18.3±11.8% vs 100%, respectively) (Fig. 2D). Similar results were observed to those found after treatment with CK666 (Fig. 2A) in which most oocytes extruded the first polar body normally in the control group (62.6%±6.5%, n = 118), but in RNAi group, oocytes underwent symmetric division (21.6±6.7%, n = 66 vs 1.4±2.5%, n = 72) (p<0.05) (Fig. 2B).

### CK666 treatment and RNAi cause failure of spindle migration and cytokinesis

**Figure 3:**
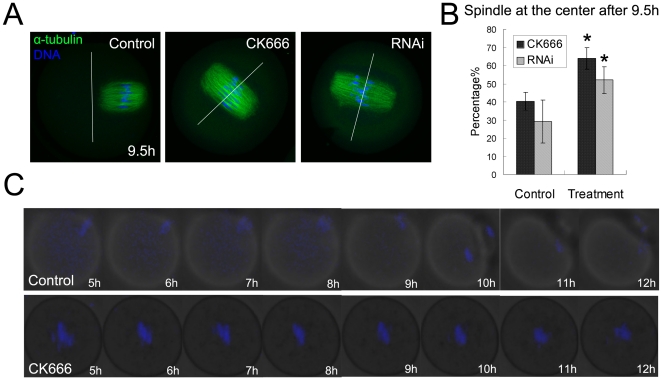
Effects of CK666 treatment and RNAi on spindle formation and migration in mouse oocytes. (**A**) In the control group, the spindle formed and moved to the cortex during the late MI stage, but remained centrally located after treatment with CK666 and RNAi. Green, α-tubulin; blue, chromatin. Bar = 20 µm. (**B**) Rate of spindle localization after 9.5 h of culture in oocytes treated with CK666 and RNAi. *, significantly different (p<0.05). (**C**) Time lapse microscope image of a maturing oocyte treated with DMSO or CK666 after GVBD. Spindle migration failed to occur in the oocyte treated by CK666.

Since the oocytes described above underwent symmetric division, the effect of the Arp2/3 complex on spindle migration was analyzed. The spindle of most of the control oocytes had formed and moved to the cortex by the late MI stage following 9.5 h of culture (Fig. 3A). However, a large proportion of those treated with CK666 (59.8±5.9%, n = 140 vs control 40.2±4.9%, n = 150; p<0.05) or RNAi (52±7.3%, n = 142 vs control 29.2±11.9%, n = 67) (Fig. 3B) possessed centrally-located spindles. Thus, the disruption of the Arp2/3 complex caused the failure of spindle migration. We then employed time-lapse microscopy to examine the dynamic changes in chromosome migration following CK666 treatment. In control group, oocyte extruded polar body normally at 12h culture; While in the CK666 treatment group, even after a 12 h culture period, the chromosomes were seen to remain in the central cytoplasm and spindle migration was disrupted, which further confirmed the involvement of the Arp2/3 complex in this process (Fig. 3C).

### CK666 treatment and RNAi cause failure of cytokinesis

**Figure 4:**
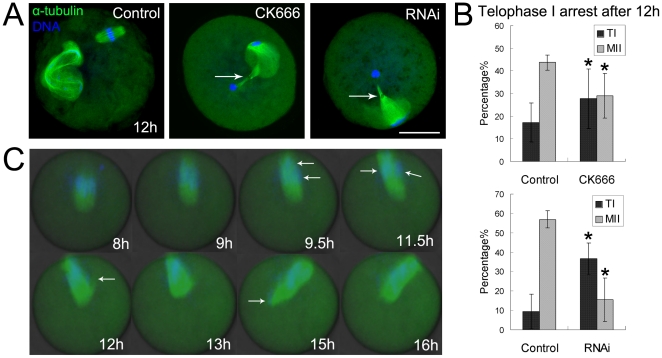
Effects of CK666 treatment and RNAi on cytokinesis in mouse oocytes. (**A**) In the control group, the oocytes extruded the polar body and arrested at the MII stage, whilst the arrest occurred during telophase I and the chromosomes condensed following treatment with CK666 and RNAi. Green, α-tubulin; blue, chromatin. Bar = 20 µm. (**B**) Frequency of telophase I arrested oocytes after 12 h in culture by RNAi. *, significantly different (p<0.05). (**C**) Time lapse microscopy of maturing oocytes treated with CK666. The oocytes failed to show the completion of cytokinesis after 16 h culture and remained at the TI stage.

After culturing for 12 h, most oocytes in the control group extruded the polar body and arrested at the MII stage, whilst most of those in CK666 treatment group arrested at telophase I and failed to extrude the polar body. Furthermore, the chromosomes were condensed by the sustained arrest (Fig. 4A) in the latter group. The rate of TI-arrested oocytes is significantly higher compared to the control group (27.7±13.2%, n = 182 vs 17.2±8.5%, n = 126), while the number of oocytes in the MII stage was significantly reduced after this treatment (29.1±9.8%, n = 182 vs 43.7±3.5%, n = 126). Similar results were observed in oocytes after RNAi (TI stage, RNAi 36.6±8.1%, n = 159 vs control 9.6±8.9%, n = 81; MII stage, RNAi 15.6±11.2%, n = 159 vs control 56.9±4.4%, n = 81) (Fig. 4B). Time-lapse microscopy showed that the chromosomes segregated at 9.5 h and reached the TI stage at 11.5 h. However, the oocytes treated CK666 remained arrested at this stage even after 16 h in culture and failed to complete cytokinesis and extrude the polar body (Fig. 4C). The results therefore indicate that disruption of the Arp2/3 complex affects completion of cytokinesis and final polar body extrusion.

### CK666 treatment and RNAi cause disruption of actin cap formation

**Figure 5:**
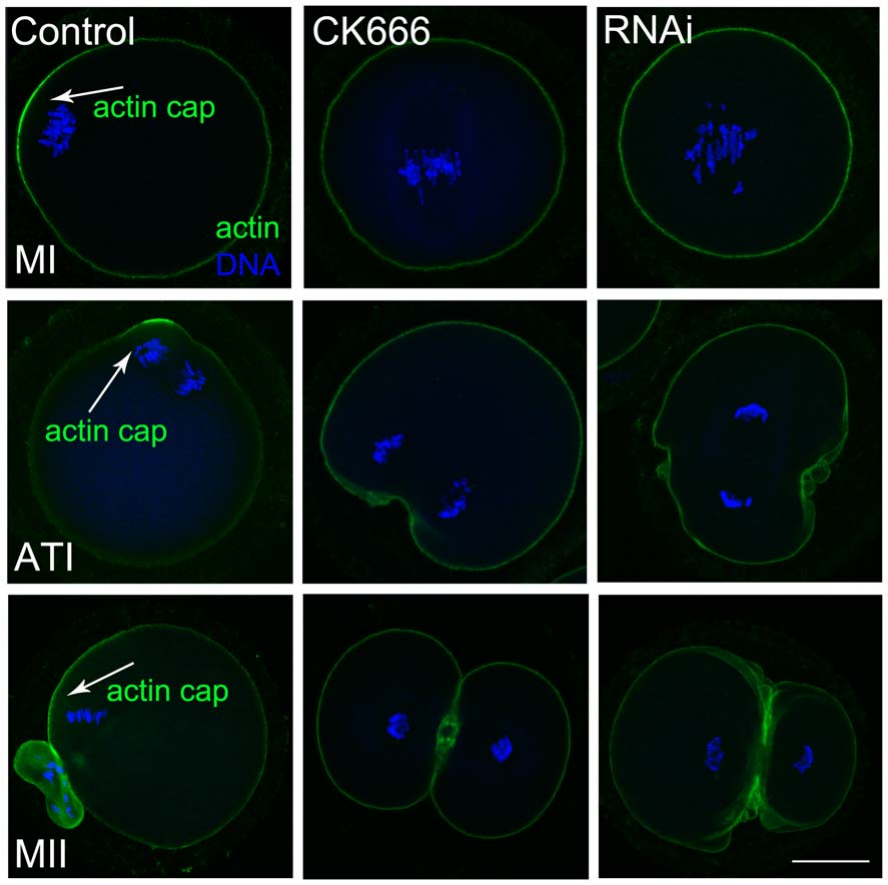
Effects of CK666 treatment and RNAi on actin cap formation in mouse oocytes. The chromosomes in the control group had moved to the cortex and an actin cap had formed by the late MI stage, whilst the chromosomes of the CK666 treatment and RNAi oocytes remained at a central position and no actin cap was observed. The chromosomes segregated at the cortex of the oocyte during TI in the control group. In oocytes treated with CK666 and RNAi, no actin cap formed, oocytes segregated with the chromosomes at the central position, and cytokinesis was initiated from this central location. During MII, a small polar body and a large oocyte formed in the control group, whilst CK666 treatment and RNAi samples formed two cell-like structures. An arrowhead illustrates the actin cap. Green, actin; blue, chromatin. Bar = 20 µm.

Symmetric division and failure of spindle migration may be due to the disruption of oocyte polarity. To assess this, we examined actin cap formation; a feature of oocyte polarization. As shown in Fig. 5, the chromosomes of the control group had already moved to the cortex and formed an actin cap by the latter part of MI, but in the CK666 treatment and RNAi group, they remained in the centre of the cytoplasm and no actin cap was observed. The chromosomes segregated at the region of the cortex with the actin cap in the control oocytes, but in the CK666 and RNAi groups, they segregated at the central position and cytokinesis was initiated. In the MII stage of the control group, a small polar body and a large MII oocyte formed, and the chromosomes were located under the region of the cortex where the actin cap had appeared. Conversely, in the CK666 and RNAi groups, the oocytes formed a 2 cell-like structure with no actin cap. Thus, actin cap formation was disrupted after CK666 treatment and RNAi.

### CK666 treatment and RNAi cause disruption of the cortical granule-free domain (CGFD)

**Figure 6:**
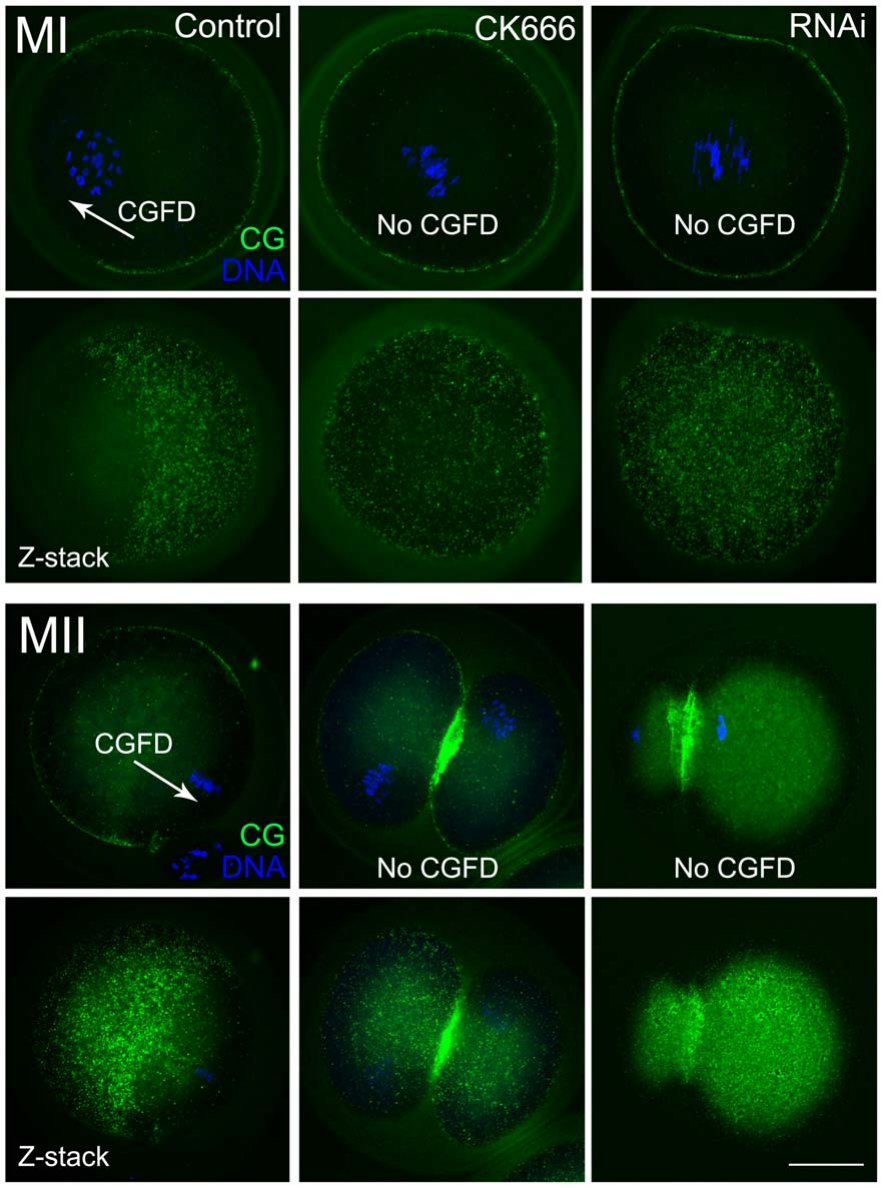
Effects of CK666 treatment and RNAi on cortical granule-free domain formation in mouse oocytes. The cortical granules were absent in the cortex close to where the chromosomes were located during the MI and MII stages in the control group. Conversely, in the oocytes treated with CK666 and RNAi, the cortical granules were distributed throughout the entire cortex. Z-stack showed the presence of different scanned layers. An arrowhead shows the cortical granule-free domain. Green, cortical granules; blue, chromatin. Bar = 20 µm.

Formation of the cortical granule-free domain (CGFD) was examined as a further feature of oocyte polarization. The cortical granules were absent near the region of the cortex close to the chromosomes during the MI stage in the control group, whilst CK666 treatment and RNAi resulted in the cortical granules being distributed uniformly across the entire cortex (Fig. 6). Similar phenotypes were observed in MII stage oocytes; CGFD formed in the MII-arrested oocytes of the control group but was absent in the 2 cell-like MII oocytes of the CK666 and RNAi groups. The results are evidence that the formation of the cortical granule-free domain was disrupted after CK666 treatment and RNAi. Taken together with the disrupted formation of the actin cap, the inhibition of CGFD formation indicates that a failure in oocyte polarization occurred.

## Discussion

The present study investigated the expression, localization, and potential functions of the Arp2/3 complex during mouse oocyte meiotic maturation. The results demonstrated the relationship between this complex and actin. In particular, the disruption of the activity of the Arp2/3 complex by specific inhibitor treatment and the RNAi approach can affect the formation of the actin cap and cortical granule-free domain, together with causing disruption to spindle migration, asymmetric division and completion of cytokinesis during mouse oocyte meiotic maturation. The study therefore provides direct evidence of the involvement of the Arp2/3 complex in oocyte polarization and cytokinesis.

### Localization of the Arp2/3 complex showed the relationship with actin in mouse oocytes

During all stages of mouse oocyte meiotic maturation, the Arp2/3 complex was mainly found alongside F-actin at the cortex and was also enriched in the region overlying the actin cap. This localization pattern is similar to that described by previous studies in somatic cells or embryo fibroblasts of other species in which the complex was showed to localize at the membrane together with F-actin [37], [38], [39], [40]. We observed the enrichment of ARP2 around the chromosomes towards the end of the MI stage, indicating that the Arp2/3 complex may be involved in the migration of chromosomes to the cortex. The complex also enriched at the area underlying the actin cap from the late MI to the MII stage, indicating that it may be involved in oocyte polarization. CB treatment disrupted the specific localization of the Arp2/3 complex that dispersed into the cytoplasm, indicating that possible relationship between the complex and actin. This observation is consistent with previous work showing that the complex regulates actin nucleation (see reviews [8], [10]). The results together suggest that the localization pattern of Arp2/3 is related to actin in mouse oocytes and indicates that the complex has a possible role on actin-related processes during oocyte meiotic maturation.

### Arp2/3 complex regulates asymmetric division through its effect on spindle migration in mouse oocytes

The roles of the Arp2/3 complex during mouse oocyte meiotic maturation were investigated using the newly developed specific inhibitor CK666 and the RNAi approach. The results demonstrated that inhibition of Arp2/3 complex activity resulted in symmetric cell division and the disruption of oocyte polarity. Multiple processes appear to be involved in the regulation of asymmetric division, the first of which is spindle positioning, including spindle migration and anchoring. The second process involves cortical reorganization and includes the loss of microvilli, cortical granule exclusion and actin cap formation [2], [41]. As the development of the actin cap and CGFD are the key steps in oocyte polarization, these events were examined in this study. Formation of both of these regions was found to be lacking after treatment with CK666 and RNAi, in contrast, actin and cortical granules were seen to be distributed uniformly throughout the cortex. These results are indicative of the disruption of oocyte polarization, which may have caused the subsequent symmetric division.

**Figure 7:**
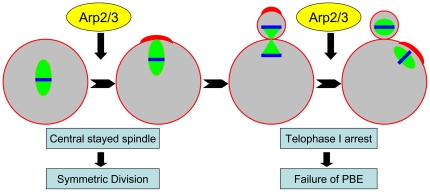
Arp2/3 complex was involved in multiple processes associated with oocyte polarization, including spindle migration and cytokinesis. Disruption of spindle migration caused the spindle to remain in a central position and symmetrical division to occur. Disruption of cytokinesis caused arrest during telophase I and a failure to extrude the polar body.

Cortical reorganization was initiated after the spindle migrated to the cortex. We therefore investigated whether the Arp2/3 complex was involved in the earlier process of spindle positioning. Previous studies have shown that Cdc42 and Rac, the activators of the Arp2/3 complex, regulate spindle formation and polar body extrusion in mouse oocytes [33], [34], [35]. Due to this link, we postulated that the Arp2/3 complex itself is also involved in this process. The results showed that the disruption of the Arp2/3 complex activity by treatment with CK666 and RNAi played a major part in the arrest of the spindle in a central location after 9.5 h in culture; a time by which the spindles of most oocytes should have moved to the cortex. Spindle migration was also dependent on actin, suggesting that the Arp2/3 complex may regulate spindle migration through its influence on actin nucleation (Fig. 7). However, disruption of the complex did not affect spindle morphology, indicating that it is probably not involved in the regulation of spindle formation itself. This finding is in contrast to results from previous work showing that spindle formation can be disrupted by deactivation of Cdc42 and Rac. We therefore suggest that Cdc42 and Rac may regulate spindle formation through a different pathway that does not involve the Arp2/3 complex. Recent work in mouse oocytes has shown that PAK1, a downstream molecule of Cdc42, can regulate spindle formation, polar body extrusion and localization of MEK in mouse oocytes [42], indicating that a Cdc42-PAK1-MEK-MAPK pathway might exist. Meanwhile, Cdc42 may activate the Arp2/3 complex through the binding of WASP, which results in the regulation of actin nucleation needed for spindle migration. The processes of actin cap formation and the subsequent formation of the CGFD would also appear to be mediated by this latter pathway.

### Arp2/3 complex regulates polar body extrusion through the effect on cytokinesis in mouse oocytes

Polar body extrusion was depended upon cytokinesis, which in turn was driven by actin. Since the Arp2/3 complex regulates actin nucleation, we speculated whether the complex also regulates cytokinesis. Our results showed that after 12 h of culture, the disruption of the activity of the complex was largely responsible for the arrested development of oocytes during telophase I, which was characterized by a failure to extrude the polar body and by the condensation of the chromosomes. These observations are indicative of a failure of cytokinesis (Fig. 7). Although Rac and Cdc42 also mediate polar body extrusion, they do this through the regulation of spindle stability and positioning. The regulation of polar body extrusion appears to be achieved using an Arp2/3 complex mechanism that is distinct from the Rac and Cdc42 mechanism. The functions of the Arp2/3 complex are similar to those described for Formin-2, JMY and cortactin [43], [44], [45] in mouse oocytes, whose disruption also results in the failure of spindle migration and cytokinesis but has no effect on actual spindle formation. This suggests that there may be a relationship between Arp2/3 and Formin-2 that would justify further investigation. In our results, some oocytes failed to migrate spindle, cytokinesis occurred in the central oocyte and 2-cell-like MII oocytes formed; some oocytes succeed in spindle migration, but were arrested at TI stage and cytokinesis failed, the controdiction may be due to the knock down efficiency and individual difference.

In conclusion, our results indicate that the Arp2/3 complex regulates oocyte polarization through wide-ranging effects on cortical reorganization, spindle migration, asymmetric division and cytokinesis during mouse oocyte meiotic maturation.

## Materials and Methods

### Antibodies and chemicals

Mouse monoclonal anti-ARP2 antibody was purchased from Abcam (Cambridge, UK), whilst Phalloidin-FITC, Lectin-FITC and mouse monoclonal anti-α-tubulin antibody were obtained from Sigma (St Louis, MO). Alexa Fluor 488 and 568 goat anti-mouse antibodies were purchased from Invitrogen (Carlsbad, CA) and CK-666 was a gift from Prof. Thomas Pollard at Yale University.

### Ethic statement

Animal care and use were conducted in accordance with the Animal Research Institute Committee guidelines of Chungbuk National University, Korea (Approval number CB-R28). Mice were housed in a temperature-controlled room with proper darkness-light cycles, fed with a regular diet, and maintained under the care of the Laboratory Animal Unit, Chungbuk National University, Korea. The mice were killed by cervical dislocation. This study was specifically approved by the Committee of Animal Research Institute, Chungbuk National University (Approval number CB-R28).

### Oocyte collection and culture

Germinal vesicle-intact oocytes were collected from the ovaries of 6- to 8-week-old ICR mice and were cultured in M16 medium (Sigma) under paraffin oil at 37°C, 5% CO_2_. Oocytes were collected for immunostaining and microinjection after a range of times in culture.

### Real-time quantitative PCR analysis

Analysis of Arpc2 and Arpc3 gene expression was measured by real-time quantitative PCR and the ΔΔC_T_ method. Total RNA was extracted from 50 oocytes using a Dynabead mRNA DIRECT kit (Invitrogen Dynal AS, Norway), and first strand cDNA was generated with a cDNA synthesis kit (Takara, Japan), using Oligo(dT)12–18 primers (Invitrogen). cDNA fragments of Arpc2 and Arpc3 were amplified using the following primers:

Arpc2,

Forward, GGAACTGAGGAGGAAGCG;

Reverse, GGAACCCAAATGGAGAAT

Arpc3,

Forward, ACAGGAGGACGAGATGAT;

Reverse, ACCACTTGCTGGGTTTAT

The DyNAmo HS SYBR Green qPCR kit (FINNZYMES, Finland) was used with a DNA Engine OPTICON 2 Continuous Fluorescence Detector (MJ Research, MA) under the following conditions: 95°C for 10 sec, and 38 cycles of 95°C for 5 sec, and 50°C for 32 sec.

### Cytochalasin B treatment

One mg/ml cytochalasin B (CB) stock was diluted in M16 medium to a final concentration of 10 µg/ml. The oocytes were cultured in this combined M16 CB medium for 8 h before collection for immunofluorescence microscopy.

### CK666 treatment

Stock CK666 (50 mM in DMSO) was diluted in M16 medium to a final concentration of 500 µM. Oocytes were then cultured in this medium for a range of times and used for immunofluorescence microscopy. The control group was cultured in the same concentration of DMSO.

### Arpc2 and Arpc3 siRNA injection

Approximately 5–10 pl of Arpc2 and Arpc3 siRNA (Ambion, TX) was microinjected into the cytoplasm of a fully-grown GV oocyte using an Eppendorf FemtoJet (Eppendorf AG, Hamburg, Germany) with a Nikon Diaphot ECLIPSE TE300 inverted microscope (Nikon UK Ltd., Kingston upon Thames, Surrey, UK) equipped with a Narishige MM0-202N hydraulic three-dimensional micromanipulator (Narishige Inc., Sea Cliff, NY). After injection, the oocytes were cultured in M16 medium containing 5 µM milrinone for 24 h, and then washed five times, each for 3 min, in fresh M16 medium. The oocytes were then transferred to fresh M16 medium and cultured under paraffin oil at 37°C in an atmosphere of 5% CO_2_ in air. The control oocytes were microinjected with 5–10 pl of negative control siRNA. The actin cap, cortical granules and spindle location were examined using confocal microscopy. Polar body extrusion was observed using a stereo-microscope.

### Time lapse microscopy

For imaging chromosome dynamics during oocyte maturation, oocytes injected with tubulin-GFP were incubated with M16 medium containing Hoechst 33342 (5 ng/ml, Sigma) and CK666. Images were taken by a 20×/0.5 objective lens (Carl Zeiss, Germany) under a computer controlled video microscope (Zeiss LSM 710 META, Germany). Exposure time was 300 ms every 20 min. The ZEN software (Carl Zeiss) was used to analyze the resulting video files.

### Confocal microscopy

To allow the single staining of ARP2, actin and CGs, oocytes were fixed in 4% paraformaldehyde in PBS for 30 min at room temperature and then transferred to a membrane permeabilization solution (0.5% Triton X-100) for 20 min. After 1 h in blocking buffer (1% BSA-supplemented PBS), oocytes were incubated overnight at 4°C or for 4 h at room temperature with 1:200 mouse anti-ARP2, 10 µg/ml Phalloidin-FITC, or 100 µg/ml Lectin-FITC. After three washes in a washing buffer (0.1% Tween 20 and 0.01% Triton X-100 in PBS), the oocytes were labeled with 1:100 Alexa Fluor 488 goat-anti-mouse IgG (for ARP2 staining) for 1 h at room temperature. The samples were co-stained with propidium iodide (PI) or Hoechst 33342 for 10 min and were then washed three times in washing buffer.

For double staining of ARP2 and actin, oocytes were stained with anti-ARP2 and Alexa Fluor 568 goat-anti-mouse IgG. They were then labeled with Phalloidin-FITC for 30 min, washed three times in PBS containing 0.1% Tween 20 and 0.01% Triton X-100 for 2 min, and stained with Hoechst 33342 (10 µg/ml in PBS) for 10 min.

The samples were mounted on glass slides and examined with a confocal laser-scanning microscope (Zeiss LSM 710 META). At least 30 oocytes were examined for each group.

### Data analysis

At least three replicates were performed for each treatment. Statistical analyses were conducted using an analysis of variance (ANOVA) and differences between treatment groups were evaluated with Duncan's multiple comparison test. Data were expressed as mean ± SEM and p<0.05 was considered to be statistically significant.
